# Effects of disc overhang diameter on the uplift bearing capacity of new-type concrete expanded-plate pile groups

**DOI:** 10.1038/s41598-024-64504-8

**Published:** 2024-06-12

**Authors:** Yongmei Qian, Mingyue Che, Da Teng, Chao Lin, Zunpeng Liu, Kai Zhang

**Affiliations:** 1 College of Civil Engineering, Jilin Jianzhu University, Changchun, 130118 China; 2 Changchun Institute of Architecture, Changchun, 130118 China; 3 Northeast Branch, China Construction Eighth Engineering Division Corp.Ltd., Dalian, China; 4 Jining Architecture Design and Research Institute, Jining, 272100 China; 5 Shandong Taikai Environmental Protection Technology Co., Ltd, Taian Shandong, 271000 China

**Keywords:** Disc overhang diameter, New-type concrete expanded plate pile (NT-CEP pile), Pile group, Uplift bearing performance, Engineering, Mathematics and computing

## Abstract

The disc overhang diameter can significantly affect the uplift bearing capacity of new concrete expanded-plate pile groups, affecting their design and practical applications. Accordingly, this effect was investigated considering the failure laws of the soil surrounding various pile types and groups. Based on the uplift bearing capacities of single and double piles, a finite element simulation was adopted to establish models for the four-, six-, and nine-pile groups. The relationship between the disc overhang diameter and uplift-bearing capacity of each pile group was explored: as the disk overhang diameter increased, the uplift-bearing capacities of the pile groups increased; however, this relationship is nonlinear. The optimal disc overhang diameter was determined as 1.5–1.75 times the pile diameter. For a constant disc overhang diameter, corner piles have a greater uplift bearing capacity than side piles in the six-pile group, and a greater uplift bearing capacity than the side and center piles in the nine-pile group. Thus, the pile-group effect depends on the pile position. The uplift bearing capacity did not increase linearly with the number of piles, and the average uplift bearing capacity of a pile in a pile group was less than that of a single pile. Therefore, the uplift bearing capacity of the pile groups decreased as the number of piles increased. The reliability of the simulation was verified via visual testing of a small-scale half-cut pile model.

## Introduction

**Figure 1:**
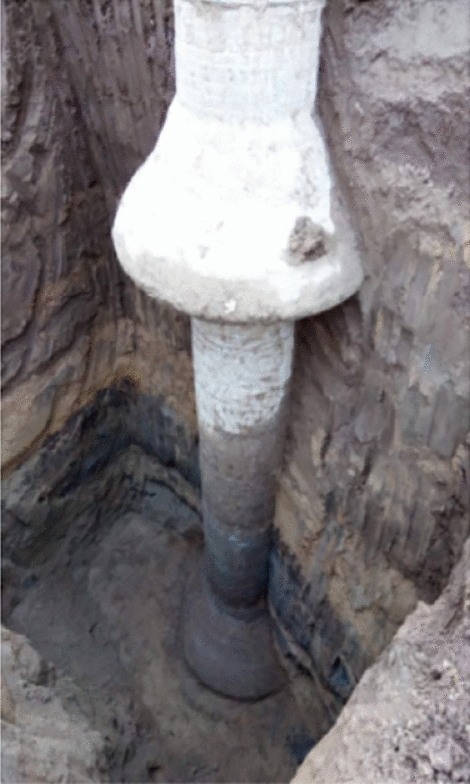
NT-CEP pile engineering diagram.

**Figure 2:**
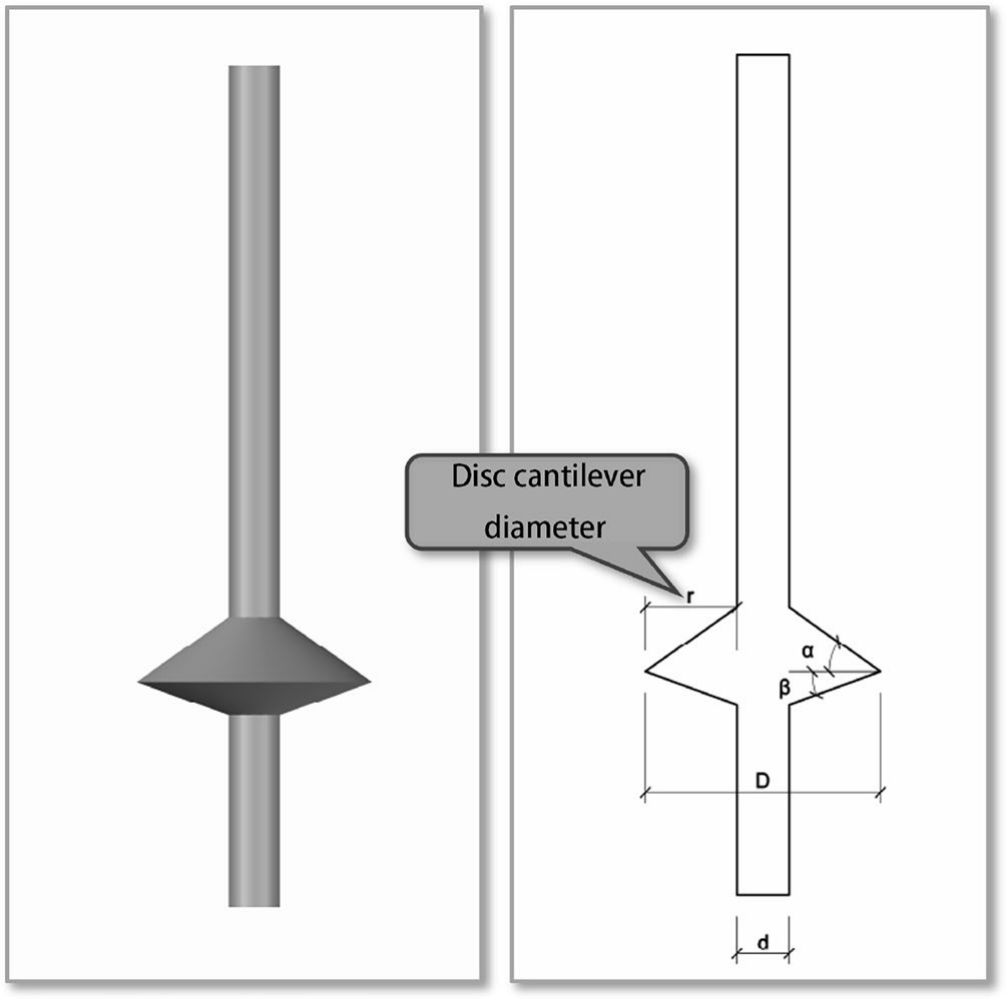
NT-CEP pile model diagram.

New-type concrete-expanded plate (NT-CEP) piles are variable-section grouting piles^1–3^ with low construction costs, high bearing capacities, and small settlements^4–6^,depicted in Figs. 1 and 2.They are different from ordinary equal diameter piles, and the ultimate tensile bearing capacity of single plate piles is 178% higher than that of equal diameter piles.Their use in engineering is increasing^7,8^; however, previous studies have mainly focused on single and double NT-CEP piles,with most studies conducted by the research group of this paper^9–11^ and few have considered pile groups^12,13^. Therefore, a systematic investigation of NT-CEP pile groups is required. Based on a project by the National Natural Science Foundation of China, the relationship between the disc overhang diameter and the uplift-bearing capacities of NT-CEP pile groups was investigated in this study.

Pile groups comprising four (2 × 2), six (2 × 3), and nine (3 × 3) piles, including corner, side, and central piles, respectively, were selected as research objects to ensure the research is representative^14^. Six ANSYS finite-element simulation models with various disc overhang diameters were established for each pile group. The pile body and soil parameters were determined based on previous research^10,15^. The stress, displacement, and failure state of the soil surrounding the piles under vertical tension were recorded using ANSYS software. The simulations for each pile group were analyzed to determine the soil damage laws in the side, corner, and central piles as well as the effects of the disc overhang diameter on the uplift bearing capacity of the pile groups. This study provides a scientific basis for improving the calculations of the uplift-bearing capacity of NT-CEP pile groups with different disc overhang diameters for practical engineering applications.

## Preliminary research

**Figure 3:**
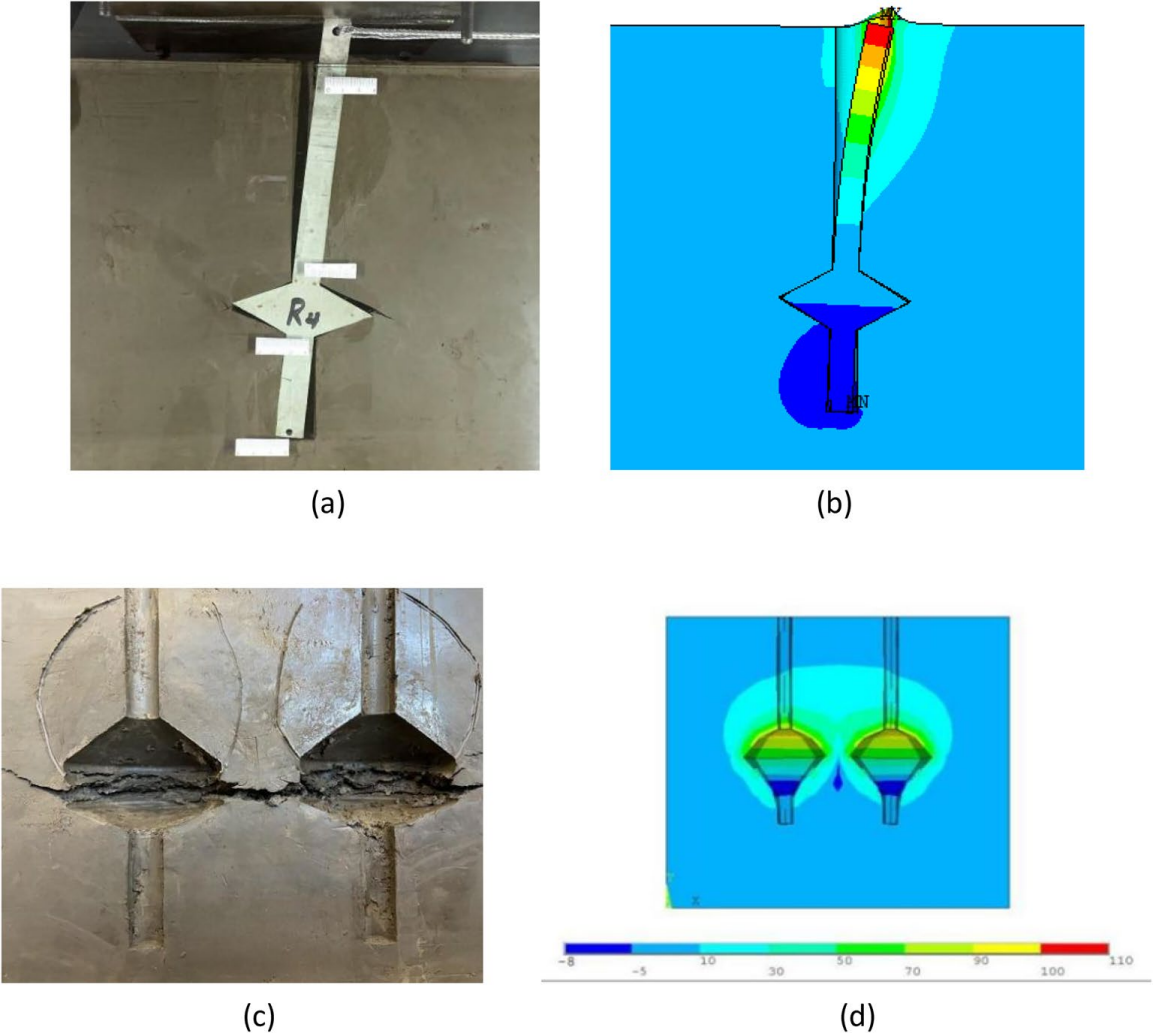
Results of previous single- and double-pile research. (**a**) Single pile under late-stage loading. (**b**) Simulation of a single pile under late-stage loading. (**c**) Soil detail map showing the failure state of a double pile. (**d**) Cloud map showing the soil displacement around a double pile under the ultimate load.

Previous studies have investigated the effects of disc overhang diameter on the uplift bearing capacity of single and double piles, and corresponding laws have been established. Research on the uplift of single and double NT-CEP piles identified various loading stages, depicted in Fig. 3.

The finite element simulations exhibit approximately the same trends as the test studies when the single- and double-pile bodies reached their ultimate loads. As shown in Fig. 3a, during the late loading period of the single-pile test, the entire pile body overturns, the upper pile body separates from the soil on the left and compresses the soil on the right, the soil around the pile is watermarked, and the lower pile body compresses the soil on the left. A cloud map of the soil displacement around the pile highlighted approximately the same region as the watermarked range in the practical test, and the trends in the pile body were approximately the same, indicating good convergence between the simulations and practical tests. Therefore, the finite element simulation of the NT-CEP pile is reliable.

As shown in Fig. 3c, when the soil around the double pile failed, horizontal cracks formed between the bearing plates of the piles, penetration occurred, and the overlying soil was compressed and deformed under the action of the bearing plates. The watermark in the soil after compression indicates that the soil was already affected. As shown in Fig. 3d, the pile body was displaced upward, and the upper parts of the load-bearing plates of the left and right piles were in contact, indicating that the piles affected each other. The conclusions from the simulation and experimental studies are approximately the same, indicating that the finite element simulation of the NT-CEP piles is reliable. Therefore, finite element simulations were employed to investigate the uplift performance of various pile groups with different disc overhang diameters in this work.

## ANSYS finite element modeling

### Constitutive relationship and element type of pile body

Based on actual engineering and preliminary experiments conducted by the research group, the pile body model is set with reference to C35 concrete parameters. The constitutive relationship of the pile body adopts an elastic–plastic model, and the yield criterion adopts the D-P criterion. The D-P criterion was chosen for numerical modeling because it has robustness when dealing with materials with different compressive and tensile strengths, and can linearly approximate the Mohr Coulomb failure envelope, simplifying the calculation process and effectively representing the general yield under combined stress. It can handle different types of soil and stress conditions with fewer parameters and a simpler calibration process, making it easy to program and perform numerical calculations. Concrete, as a non-homogeneous material, has many factors that affect its mechanical properties, and its mechanical properties exhibit significant variability in experiments. In order to consider these nonlinear factors, it is often necessary to introduce many interrelated parameters in practical applications. Therefore, the type of pile body element used in this article is Solid65, a 3D solid element. Solid65 element is defined as an eight node isotropic element, with each node having three degrees of freedom for translation in the x, y, and z directions. Solid65 is based on Solid45 and adds material parameters for concrete, which has better simulation effects on the cracking, creep and other characteristics of concrete in practical engineering^16^.

### Material parameters

The parameters of the pile body and soil material are determined based on actual engineering and the preliminary test results of the research group, and are consistent with those of our previous study. The CEP pile body was made of C35 concrete, and the simulated material properties are listed in Table 1.

**Table 1:** Material property parameters of simulated pile-soil.

Material	Density (t/mm^3^)	Elastic modulus (MPa)	Poisson’s ratio	Cohesive (kPa)	Friction angle (°)	Pile–soil friction coefficient
Concrete	2.25 × 10^–9^	3.15 × 10^4^	0.2	–	–	0.3
Clay	1.688 × 10^–9^	25	0.35	43.55	10.7	

### Model parameters

**Figure 4:**
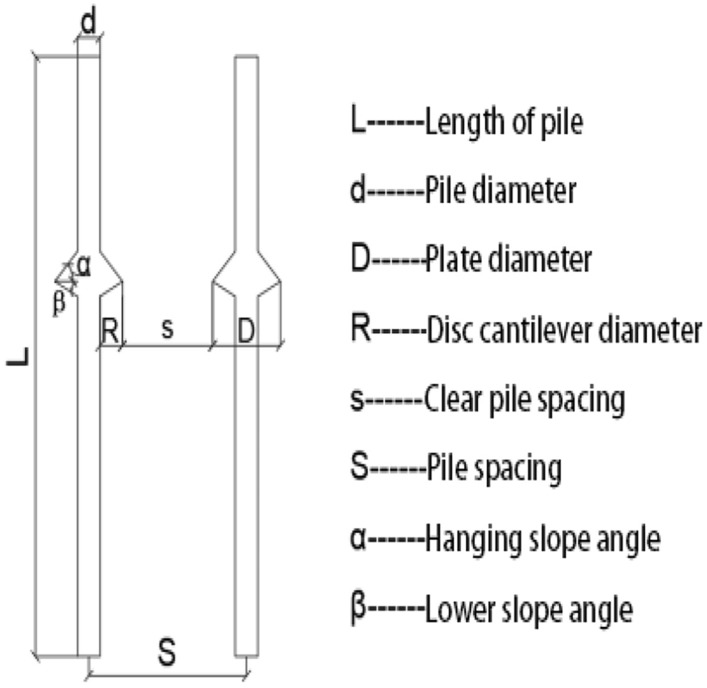
Schematic of model piles.

**Figure 5:**
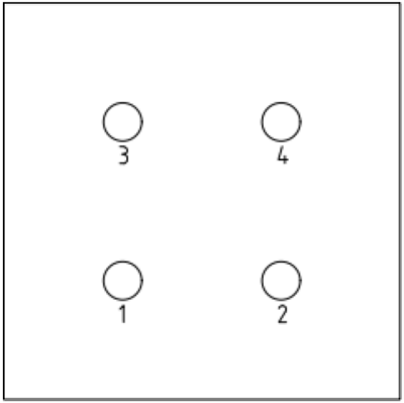
Four-pile layout.

**Figure 6:**
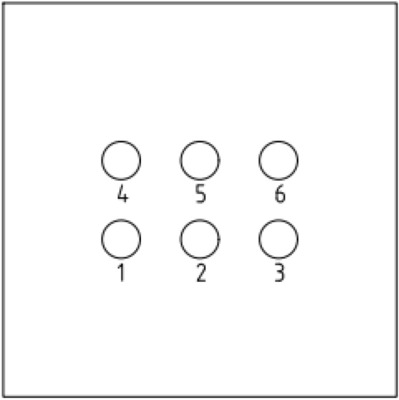
Six-pile layout.

**Figure 7:**
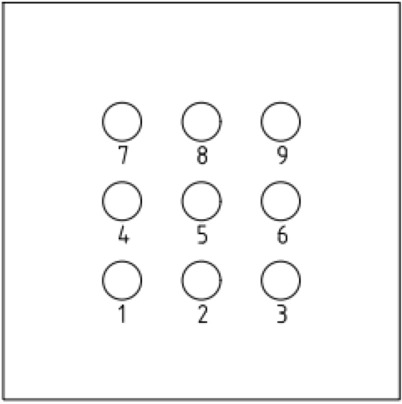
Nine-pile layout.

To increase the accuracy of the simulation, a finite element model was created on a 1:1 scale. The pile body and soil parameters were determined according to previous studies on single and double piles. The length and diameter of the pile body were 8 and 0.5 m, respectively. The bearing disk was asymmetric according to the NT-CEP pile construction technology, and the upper and lower slopes of the disk were 35° and 20°, respectively. The pile spacing was 3.5 m. Six sets of simulation studies were conducted for four (2 × 2), six (2 × 3), and nine (3 × 3) piles with disc overhang diameters of 1.0, 1.25, 1.5, 1.75, 2.0, and 2.25 times the pile diameter *d*, labeled as MG1–MG6, respectively. The pile body was located 4 m from the soil edge to reduce the influence of boundary effects on the simulation. Figure 4 presents a schematic of the model structure, and Figs. 5, 6, 7 respectively show the four-, six-, and nine-pile layouts.

### Model construction and loading scheme

**Figure 8:**
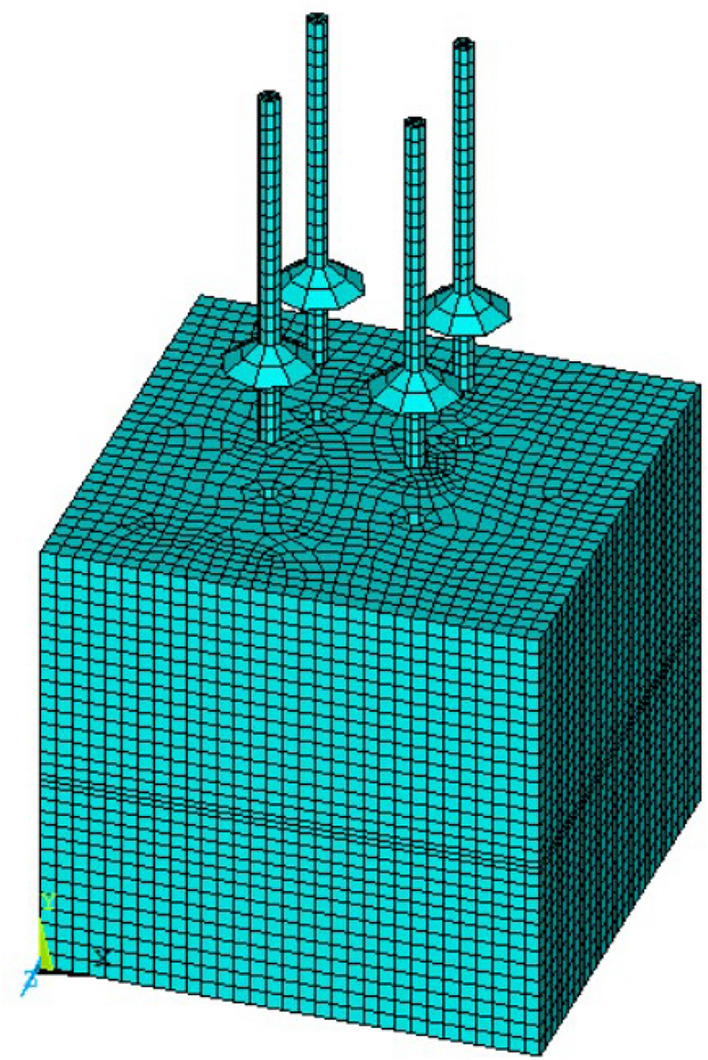
Model grid division.

This simulation analysis adopts the method of mapping mesh division^17,18^. The finite mesh shape rules generated by this method are much fewer than the corresponding free mesh, which can greatly save calculation time and ensure the accuracy and precision of the calculation results. The divided pile soil model is shown in Fig. 8.

### Pile soil contact

Owing to the differences between the material properties of the pile and soil, a large shear stress is generated when there is a relative displacement between them. Therefore, the following assumptions were made when studying the interactions between the piles and the soil:

1. Throughout the simulation, the pile side coefficient of friction remained constant, and the soil material in the finite element model was isotropic. When the pile reached its ultimate load, the soil was destroyed; however, the body of the pile remained intact.
2. The soil boundary range was significantly greater than ten times the pile diameter, and the effects of the boundary constraints on the simulation were ignored^19,20^.
3. The soil around the pile was regarded as an elastic–plastic material, and the Drucker–Prager yield criterion was applied.

**Figure 9:**
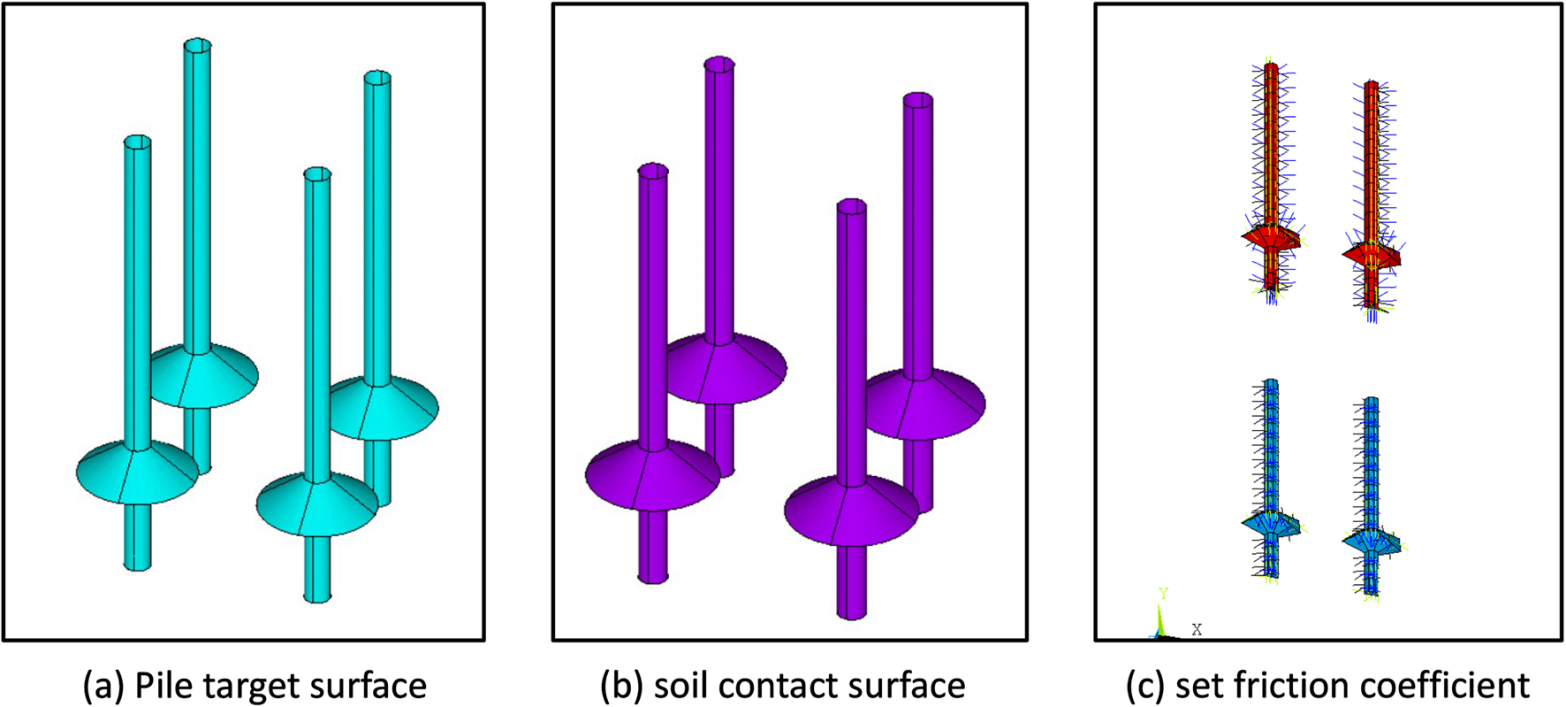
Pile soil contact analysis.

Due to the fact that the elastic modulus of the NT-CEP pile body is much greater than that of the surrounding soil, the contact surface between the pile body and the soil is set as a rigid flexible contact form. During the loading process, the pile body does not undergo damage, and the outer surface of the pile body is set as a rigid surface. The target surface is defined as the target170 element in ANSYS software, as shown in Fig. 9a. The target170 element describes the geometric shape and material characteristics of the pile body. It is a nonlinear, bending stiffness distributed pile element suitable for simulating the deformation and stress distribution of the pile. Define the contact surface between the soil and the pile body using the contact173 element, as shown in Fig. 9b. Unit contact173 describes the frictional force and contact pressure between the pile soil contact surfaces. This unit can consider the non-linear characteristics of the contact surface, including frictional slip, normal stiffness, and changes in contact area. To simulate the lateral friction resistance between the pile body and the soil around the pile in actual engineering, the selected target surface and contact surface were set with a friction coefficient of 0.3, as shown in Fig. 9c.

### Constraint settings

**Figure 10:**
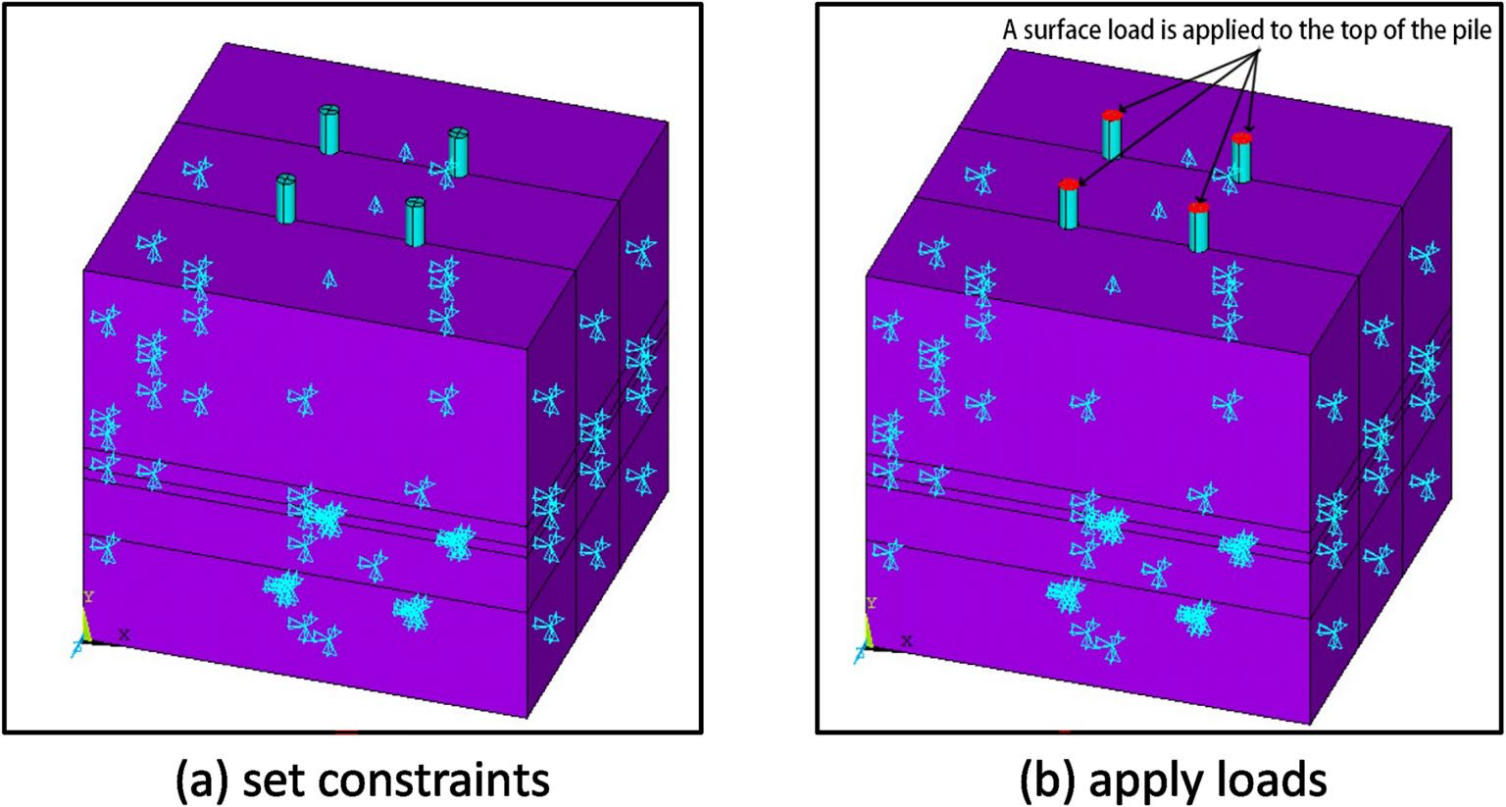
Exert restraint and load.

Because the size range of the soil outside the pile body is much larger than the influence range of the pile body, the influence of the boundary effect of the soil body on the results can be ignored.In real engineering scenarios, the pile body forms the upper part of the pile, and the soil body on top of the pile body limits the vertical movement. Therefore, in the simulation, movement in the *y* direction was constrained by the top of the soil body. The remaining surfaces exhibited limited movements in all three directions, as shown in Fig. 10a after setting the constraints.

### Apply load

To ensure that the simulation results were accurate and consistent with actual scenarios, a surface load was applied at the top of the pile body to represent the vertical tension on the NT-CEP pile.Simulate and analyze the loading process by grading the load size, and extract the load displacement data. After applying the load, as shown in Fig. 10b, refer to the conclusions of the previous single pile and double pile foundation pull-out tests of the research group, as well as the technical specifications for building foundation pile detection, building foundation treatment, and building foundation pile technology. Control the load size and load it step by step until the conditions for terminating loading are reached. The conditions for terminating loading include:

1. Under a certain level of load, the displacement of the pile top is 5 times greater than the displacement under the previous level of load;
2. Control according to pile top displacement, when the cumulative pile top displacement exceeds 100 mm;
3. According to the tensile strength control of steel bars, the load should reach 0.9 times the standard value of steel bar strength;
4. For engineering piles subjected to acceptance sampling inspection, the maximum uplift load required by the design shall be achieved.

## Analysis of ANSYS finite element simulation

### Four-pile displacement cloud map analysis

**Figure 11:**
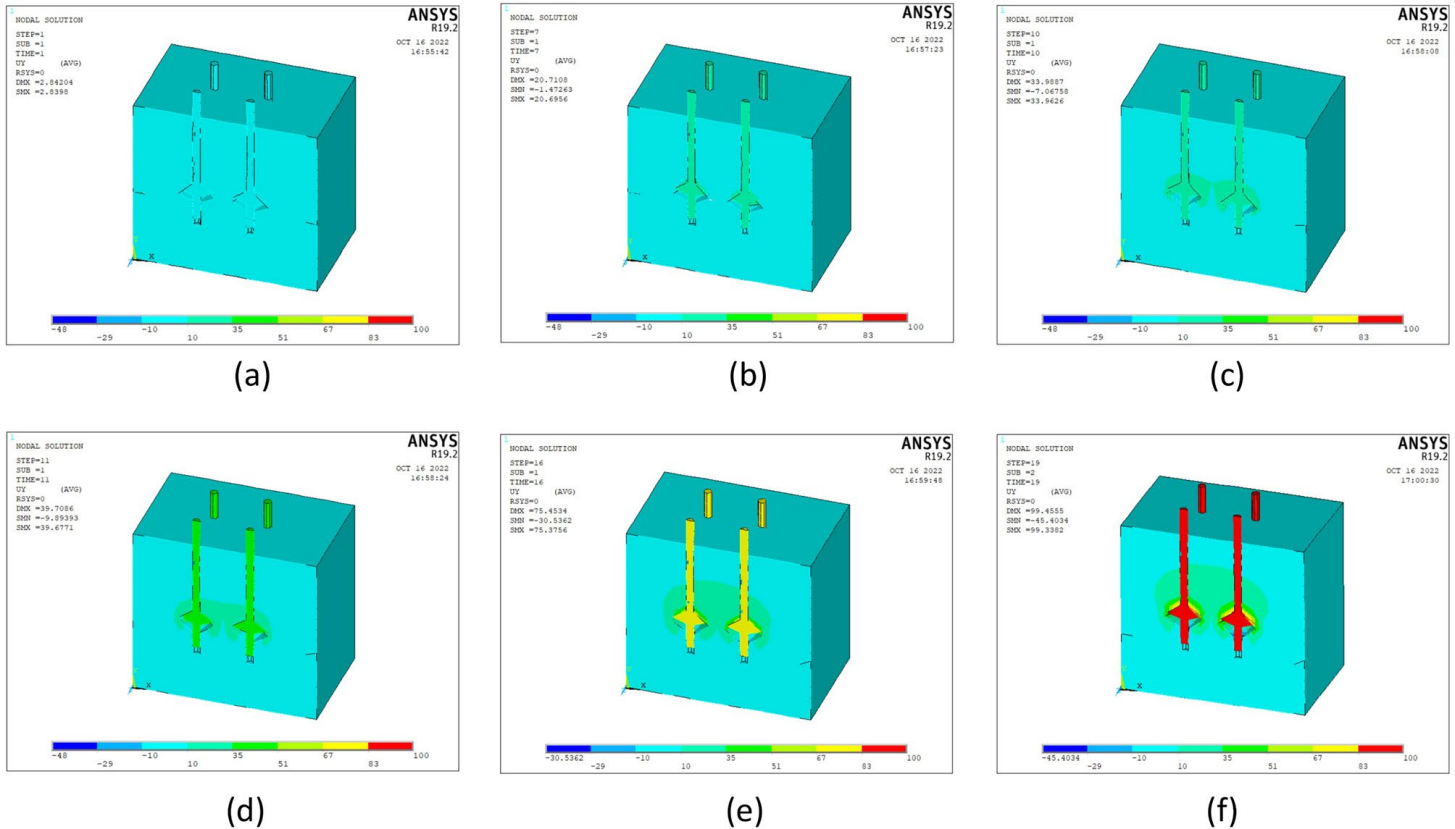
Pile–soil displacement cloud maps for the four-pile model under loads of (**a**) 400, (**b**) 2800, (**c**) 4000, (**d**) 4400, (**e**) 6400, and (**f**) 7600 kN.

Vertical tension was applied to four pile models with six different disc overhang diameters. Load control was adopted, and the displacement data were tracked and extracted simultaneously. Displacement cloud maps were observed to accurately determine changes in the pile body and surrounding soil under different loads. Group MG4 exhibited the clearest pile pattern; therefore, it was used for further analysis. The *y*-direction displacement cloud maps were extracted for the vertical tensions of 400, 2800, 4000, 4400, 6400, and 7600 kN, as displayed in Fig. 11.

The cloud maps show that:

1. During the initial loading stage, when the load was 400 kN (Fig. 11a), the bearing disc separated from the underlying soil. The bearing capacity was primarily provided by the lateral friction resistance of the pile, and the bearing disc played a minor role. When the applied load was increased to 2800 kN (Fig. 11b), the bearing disc compressed the overlying soil and began to affect the bearing capacity.
2. When the load was increased to 4000 kN (Fig. 11c), the soil masses between the four piles began to interact. However, the soil mass displacement between the piles was small and had little effect on the bearing performances of the four piles. When the load increased to 4400 kN (Fig. 11d), horizontal cracks formed between the piles, and soil slip occurred on the discs. Thus, an approximately inverted core-type displacement cloud image was obtained.
3. When the load was increased to 6400 kN (Fig. 11e), the soil slipping phenomenon on the disc became more pronounced, and the soil interaction between the four piles increased. When the load reached 7600 kN (Fig. 11f), the soil interaction between the piles increased, the displacement cloud image of the upper part of each bearing disc exhibited a large-scale contact phenomenon, and the vertical displacement data of the pile top reached its ultimate displacement.

In summary, as the vertical tension increased, the compression of the overlying soil by the bearing disk and the effects of the four piles on the soil increased.

### Six-pile displacement cloud map analysis

**Figure 12:**
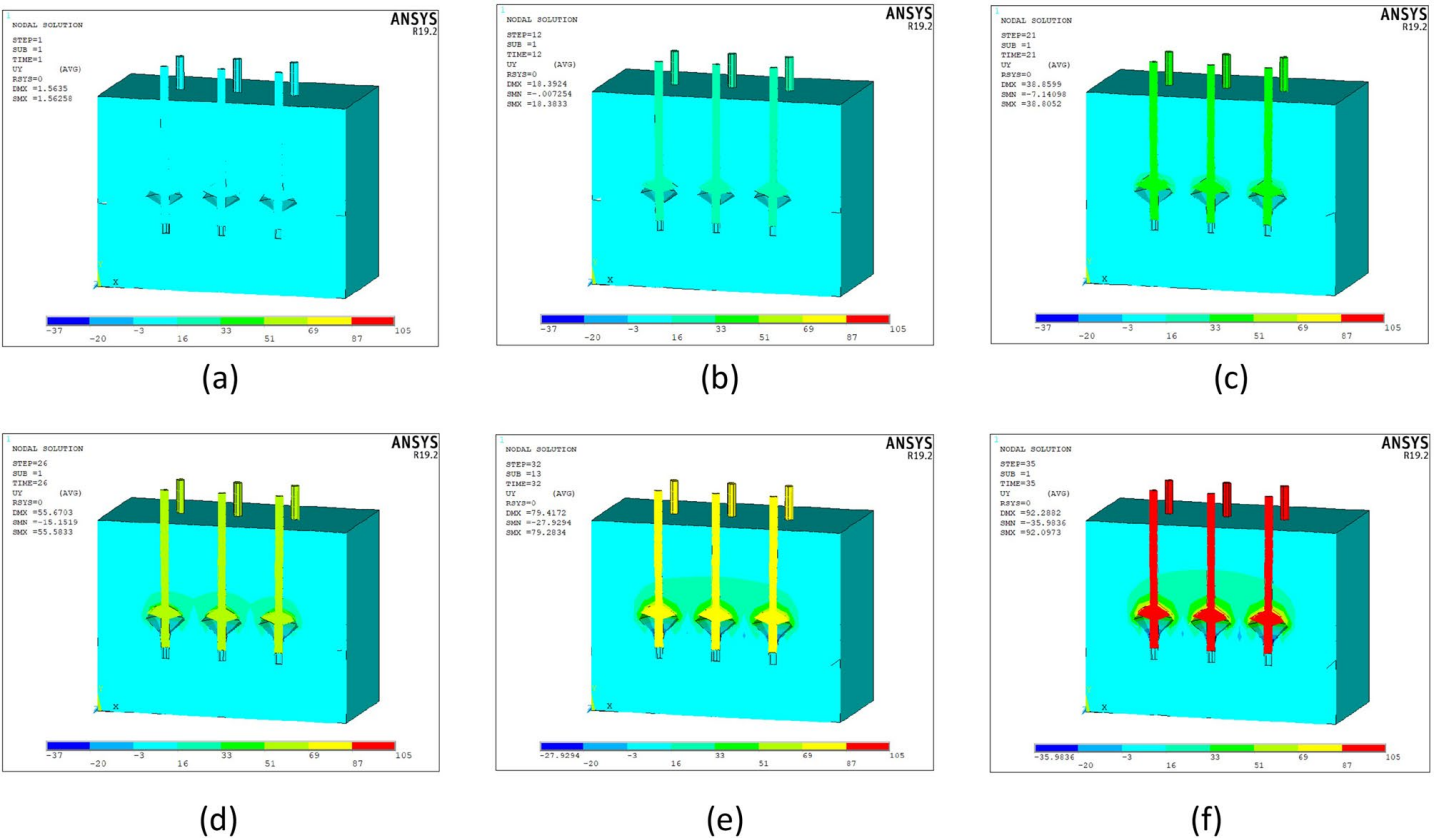
Pile–soil displacement cloud maps for the six-pile model under loads of (**a**) 300, (**b**) 3600, (**c**) 6400, (**d**) 7800, (**e**) 10,500, and (**f**) 10,500 kN.

Displacement cloud maps were used to accurately observe changes in the pile body and surrounding soil under different loads. Group MG4 exhibited the clearest pile pattern; therefore, it was considered for further analysis. The *y*-direction displacement cloud maps were extracted for vertical tensions of 300, 3600, 6400, 7800, and 10,500 kN, as presented in Fig. 12.

The cloud maps show that:

1. During the initial loading stage, when the load was 300 kN (Fig. 12a), the bearing disc separated from the soil. The bearing capacity was primarily provided by the pile-side friction resistance, and the bearing disc played a minor role. When the applied load was increased to 3600 kN (Fig. 12b), the displacement in the area near the pile body increased, and the bearing disc compressed the overlying soil and began to affect the bearing capacity.
2. When the load increased to 6400 kN (Fig. 12c), the compression of the overlying soil increased, and the soil began to slip. When the load was increased to 7800 kN (Fig. 12d), the soil between the piles began to interact. Under the same load, the displacement at the bearing plate of the side pile exceeded that at the corner pile.
3. When the load was increased to 10,500 kN (Fig. 12e), the soil interactions between the piles increased. When the load was increased to 10,500 kN (Fig. 12f), the soil interaction between the piles increased, the displacement cloud image of the upper part of each bearing disc showed a large-scale contact phenomenon, and the six piles mutually affected each other.

In summary, as the vertical tension increased, the compression of the overlying soil by the bearing disk and the interaction between the six piles increased. The displacements of the side piles were slightly larger than those of the corner piles.

### Nine-pile displacement cloud map analysis

**Figure 13:**
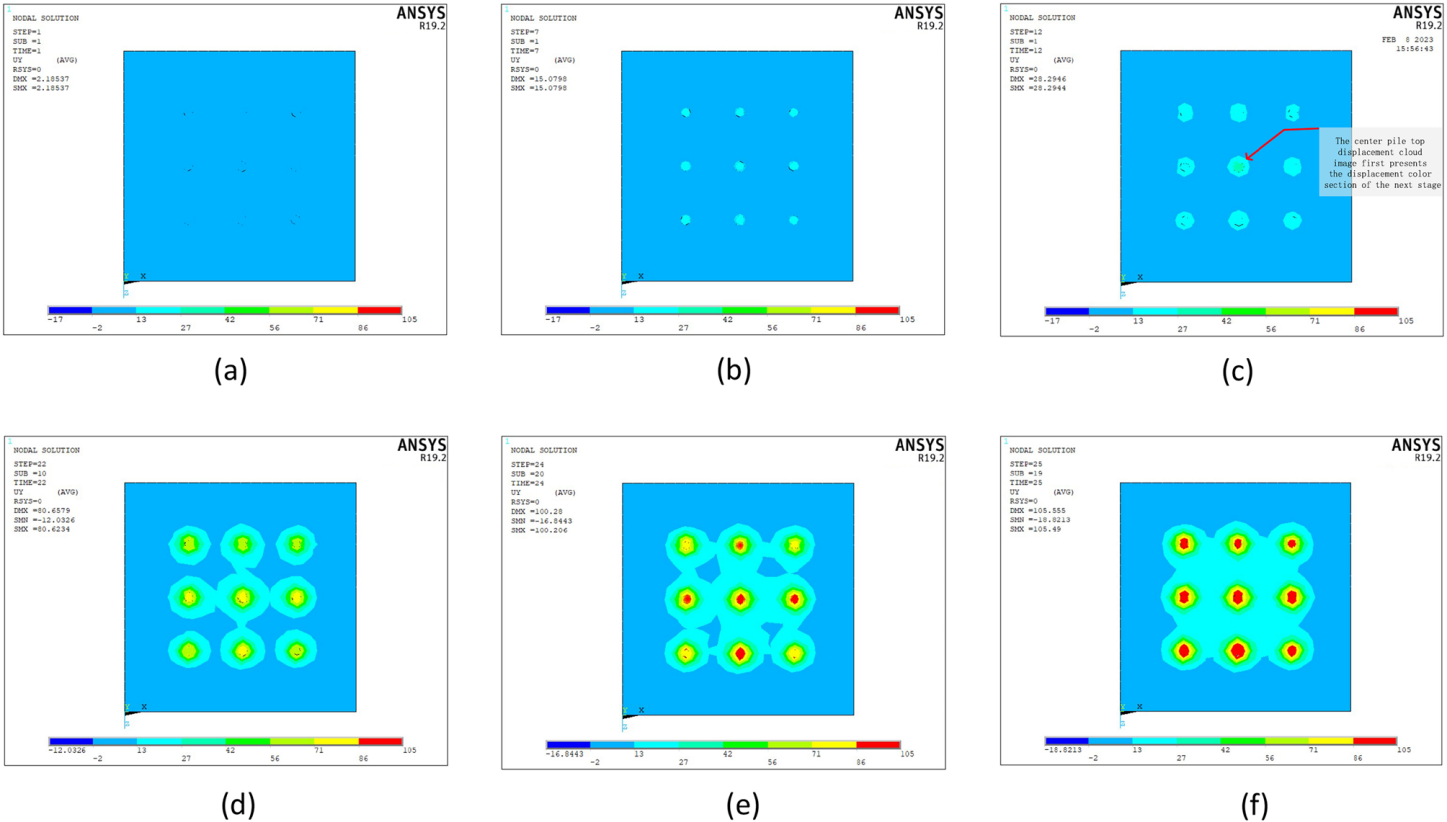
Displacement cloud maps for the top surface of the nine-pile model under loads of (**a**) 450, (**b**) 3150, (**c**) 5400, (**d**) 9900, (**e**) 10,800, and (**f**) 11,250 kN.

The nine-pile model contained more center piles than the six-pile model, and the number of corner panels was the same. Therefore, cloud maps of the nine piles were considered to study the displacement and stress changes in the pile bodies and surrounding soil under different loads. Owing to the large number of piles, group MG2 exhibited the clearest pile pattern; therefore, it was used for further analysis. The displacement cloud maps for the top surface were extracted when the vertical tensions were 450, 3150, 5400, 9900, 10,800, and 11,250 kN, as shown in Fig. 13.

Owing to the differences between the six- and nine-pile models, the effect of the center pile on the displacement cloud map of the top surface of the pile body increased, and the time-sequence effects of the center, side, and corner piles were considered.

The cloud maps show that:

1. During the initial loading stage, when the loads were 450 and 3150 kN (Fig. 13a and b), the range of displacement matched the size of the pile body. The entire pile body exhibited a small upward displacement. The bearing disc played a minor role, and the bearing capacity of the pile body was primarily provided by the pile-side friction resistance. When the load increased to 5400 kN (Fig. 13c), the displacement of the top surface of the pile body increased, and the bearing disc compressed the overlying soil and began to affect the bearing capacity. In the displacement cloud image, the color surrounding the central pile corresponds to the next stage, indicating that the displacement was larger than that of the other piles under the same load.
2. When the load was increased to 9900 kN (Fig. 13d), the bearing disc continued to compress the overlying soil. The displacement of the center pile increased the fastest, followed by that of the side piles, while the displacement of the corner piles increased the slowest. The regions displaced by the center and side piles were in contact, indicating that the piles affected each other. The displacement of the center pile was the largest, followed by that of the side piles, and the displacement of the corner piles was the smallest. When the load was increased to 10,800 kN (Fig. 13e), the regions displaced by the nine piles were in full contact. In the displacement cloud image, the color between the central and side piles corresponds to the next stage, indicating the formation of horizontal cracks in the soil. The nine piles affected each other, and the displacements of the central and side piles exceeded those of the corner piles.
3. When the load increased to 11,250 kN (Fig. 13f), the displacement of the top surface of the pile body exceeded 95 mm, and the pile body reached the ultimate load state.
4. In summary, as the vertical tension increased, the compression of the overlying soil by the bearing disk and the interaction between the nine piles increased. The center pile exhibited the greatest displacement, followed by the side piles, and the corner piles exhibited the least displacement.

Further analysis of the shear stresses and uplift bearing capacities of the center, side, and corner piles is required.

### Shear stresses of the corner, side, and central piles of the nine-pile model under the ultimate load

**Figure 14:**
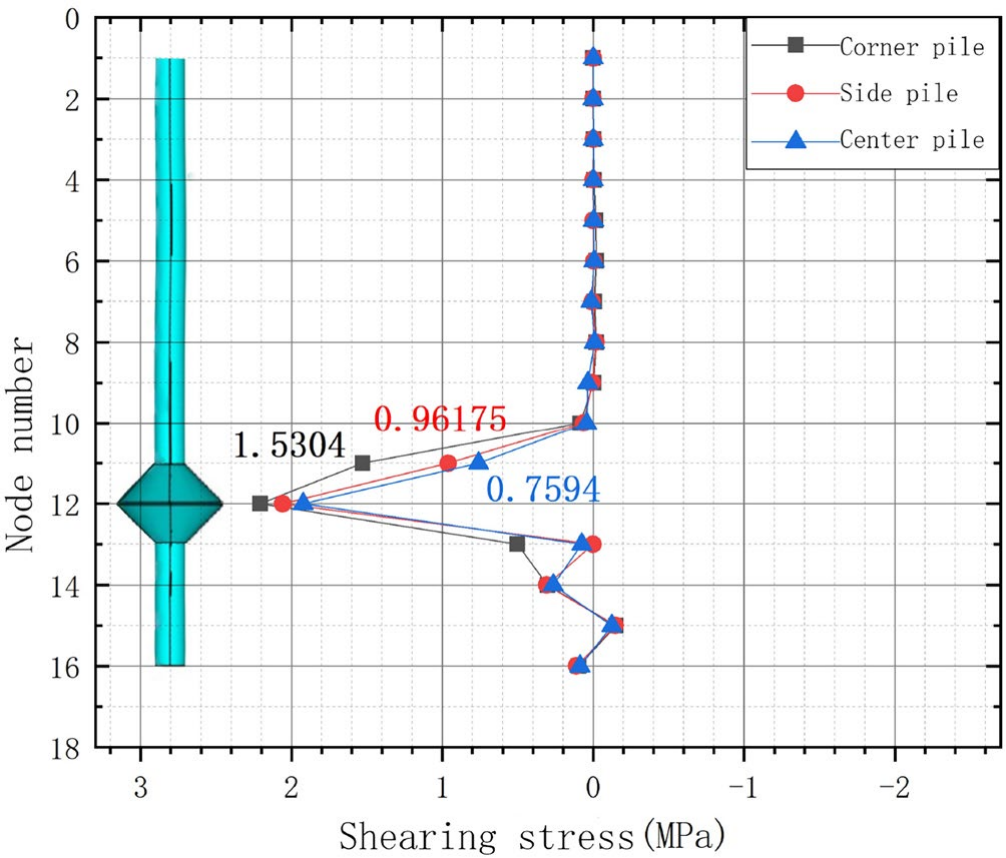
Shear stress curves for the left of the corner, side, and center piles in the nine-pile model under the ultimate load.

Considering the symmetry established in the nine-pile model, one quarter of the pile–soil model was considered in the comparative analysis of the simulated piles. The XY shear stress data for several nodes in the middle, corner, and side piles of MG2 under vertical tension were extracted, and the shear stress curves are presented in Fig. 14.

Figure 14 shows that under the ultimate load, the shear stress curves of all three piles increased and then decreased. The magnitude of the shear stress differed only between the piles at the bearing disc position and was approximately the same for the other nodes. The values fluctuated slightly by approximately 0 MPa, indicating that the bearing disc plays a major role in resisting vertical tension. The shear stresses at node 11 in the left corner, side, and central piles were 1.5304, 0.96175, and 0.7594 MPa, respectively. The corner pile exhibited the largest shear stress, followed by the side pile, and the central pile exhibited the lowest shear stress. This indicates that there were interactions between the nine piles under a vertical tensile force. The central pile was the most affected by these interactions, and its uplift-bearing capacity was the weakest.

In summary, the corner piles had the highest pulling capacity, followed by the side piles, and the central pile had the lowest pulling capacity.

### Load–displacement curves for the four-pile model

**Figure 15:**
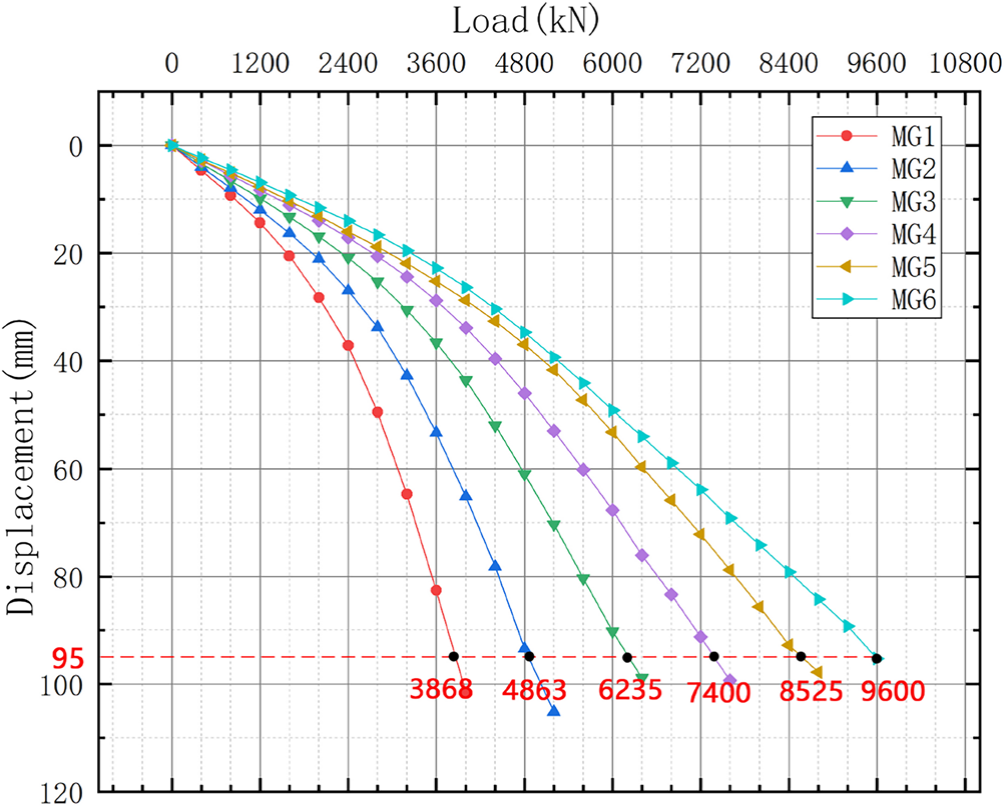
Load–displacement curves for the four-pile models with different disc overhang diameters.

**Figure 16:**
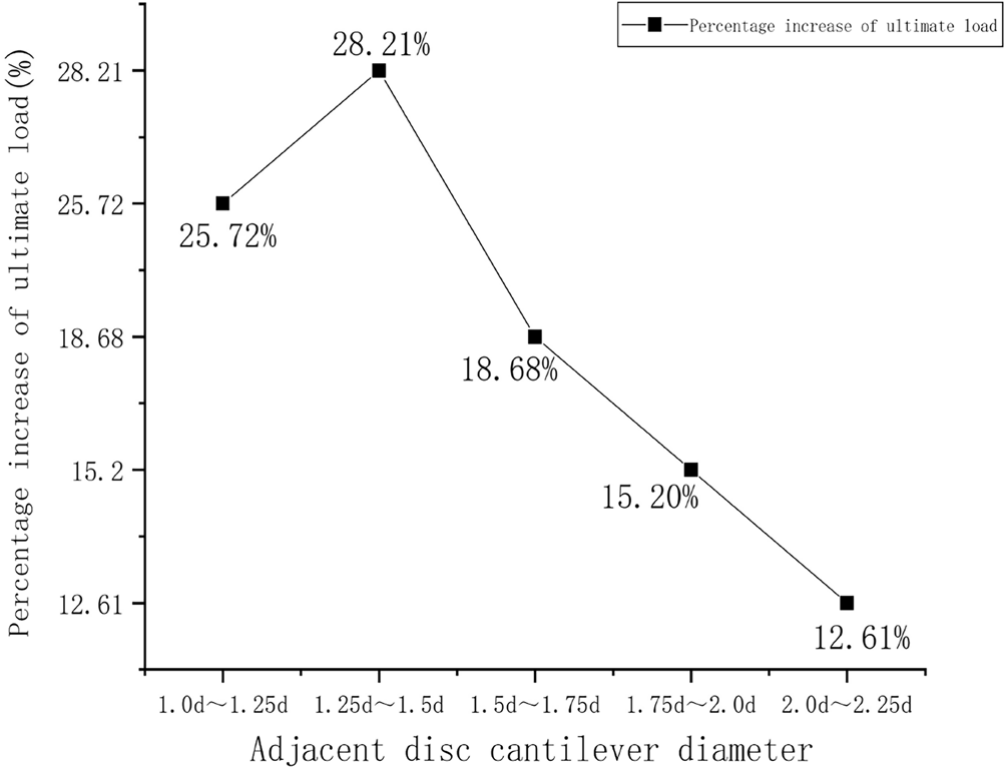
Percentage difference between the ultimate loads of models with adjacent disc overhang diameters.

The load–displacement data were extracted for the six four-pile models with different disc overhang diameters from ANSYS finite element simulation software, and the load–displacement curves were constructed accordingly, as shown in Fig. 15. When the displacement of the pile top reaches 95mm, the soil around the MG6 pile group reached a state of failure, and the MG6 group is the first to reach the ultimate load. Six sets of simulated pile ultimate load values are extracted, and the percentage difference in the ultimate load of adjacent plate cantilever diameter is shown in Fig. 16.

Under a constant load, the vertical displacement of the piles and the carrying capacity increased as the disc overhang diameter increased. During the initial loading stage, the curve gradually decreases. However, as the vertical tension increases, the curves begin to decrease more rapidly. A comparison of these results with the displacement cloud map shows that the load–displacement curve did not decrease rapidly when the overlying soil began to compress. The first horizontal cracks began to form at the edge of the disc, and there was no soil interaction between the piles. As the load increased, the load–displacement curve decreased more sharply as the horizontal cracks connected and the soil between the piles was strained and damaged, which reduced the bearing capacity.

Figure 16 indicates that the load difference was greatest when the disc overhang diameter was 1.25*d*–1.5*d*, when the load required for the pile body to reach the limit state increased by 28.21% and the bearing capacity of the pile group increased considerably. With a further increase in the disc overhang diameter, the load difference gradually decreased, and the improvement in the bearing capacity of the pile group gradually decreased. This indicates that it is necessary to select a disc overhang diameter within a reasonable range to improve the bearing capacity effectively.

### Load–displacement curves for the six-pile model

**Figure 17:**
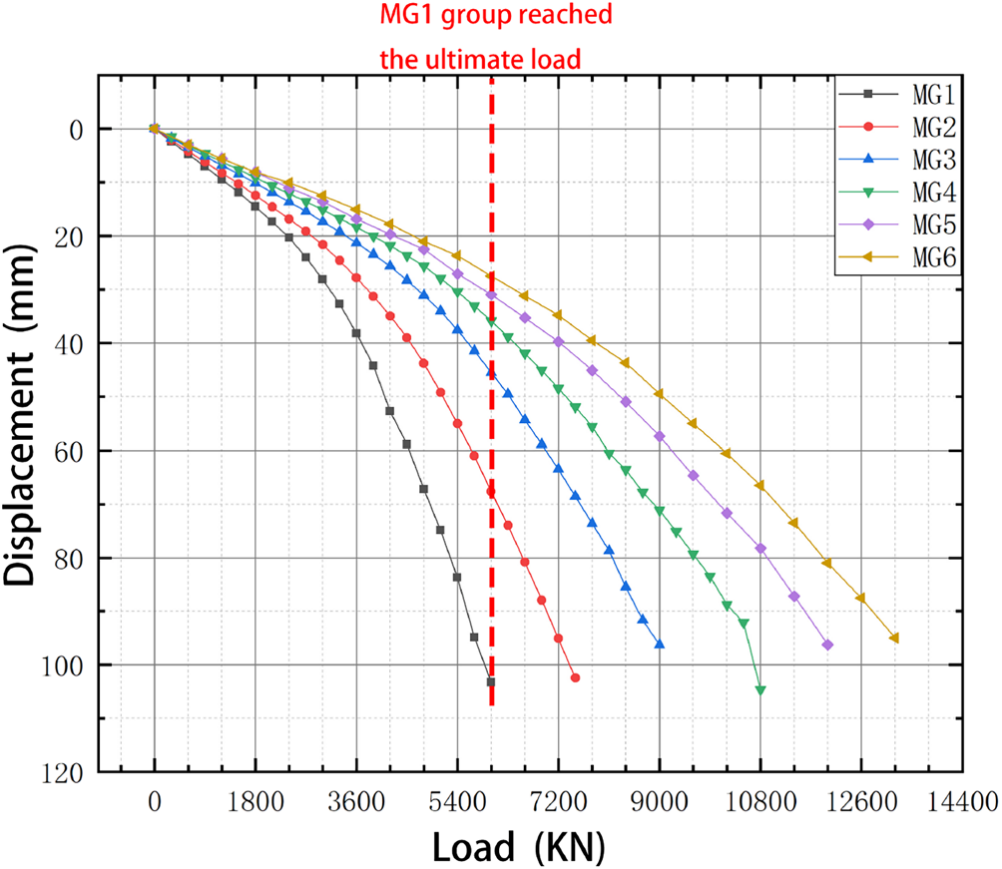
Load–displacement curves for the six-pile models with different disc overhang diameters.

**Figure 18:**
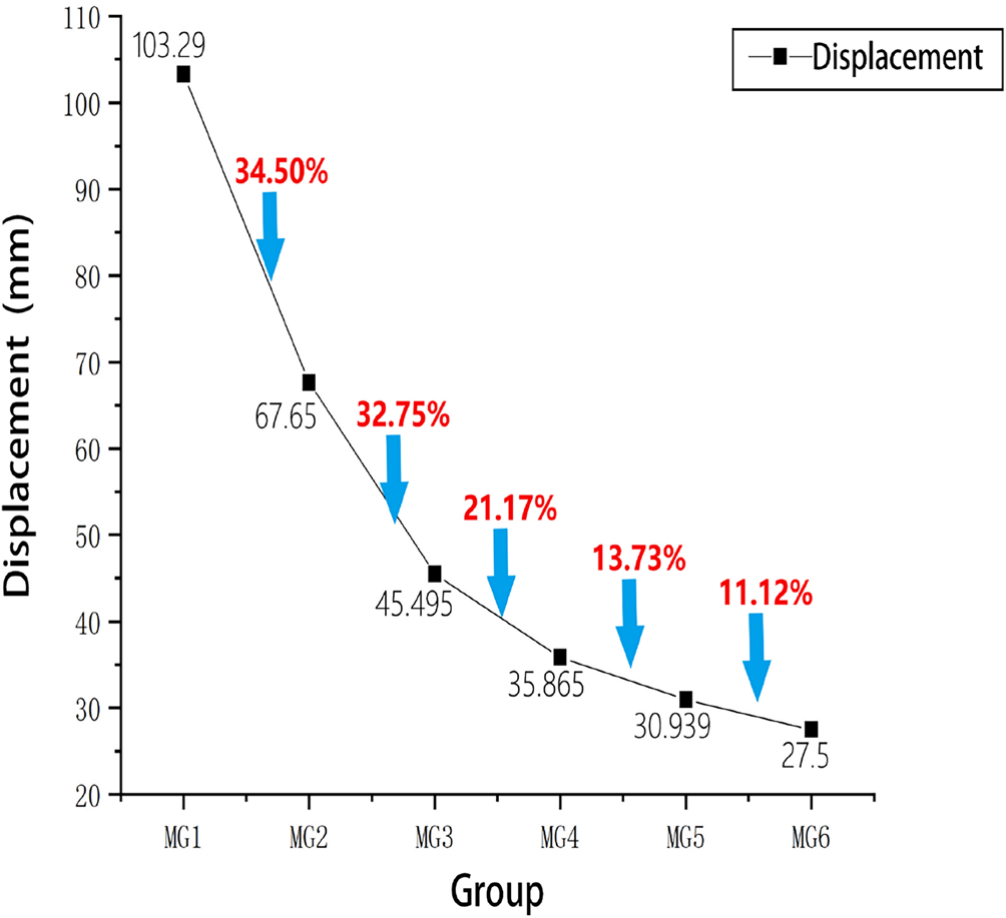
Displacement-change curves for the six-pile models with different disc overhang diameters under the same load.

The load–displacement data were extracted for the six six-pile models with different disc overhang diameters from ANSYS finite element simulation software, and the load–displacement curves were constructed, as shown in Fig. 17. Pile displacements were extracted at 6,000 kN when MG1 reached the ultimate load state. The displacement-change curves for the models with different disc overhang diameters under the same load are shown in Fig. 18.

Figures 17 and 18 show that, under a constant load, the vertical displacement of the pile body decreased as the disc overhang diameter increased, which corresponds to an increase in the uplift bearing capacity. However, the increase in uplift capacity was not linear. As the disc overhang diameter increased, the rate of change in the displacement gradually decreased. As shown in Fig. 18, the uplift-bearing capacity of the pile group decreased gradually. When the disc overhang diameter was greater than or equal to 1.75*d* (i.e., groups MG4–MG6), there was little change in the soil displacement near the piles, and the uplift-bearing capacity of the pile groups did not significantly increase as the disc overhang diameter increased. This indicates that the rate of increase in the uplift-bearing capacity decreases as the disc overhang diameter increases. Therefore, the ratio of the disc overhang diameter to pile diameter must be within an appropriate range.

### Load–displacement curves for the nine-pile model

**Figure 19:**
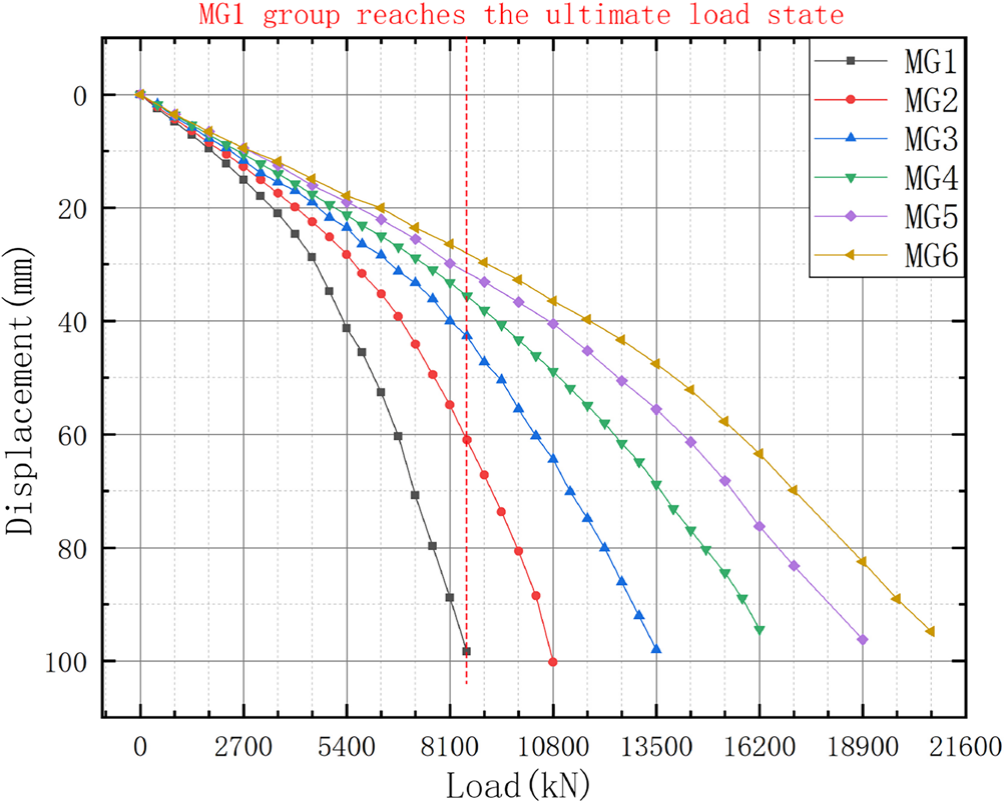
Load–displacement curves for the nine-pile models with different disc overhang diameters.

**Figure 20:**
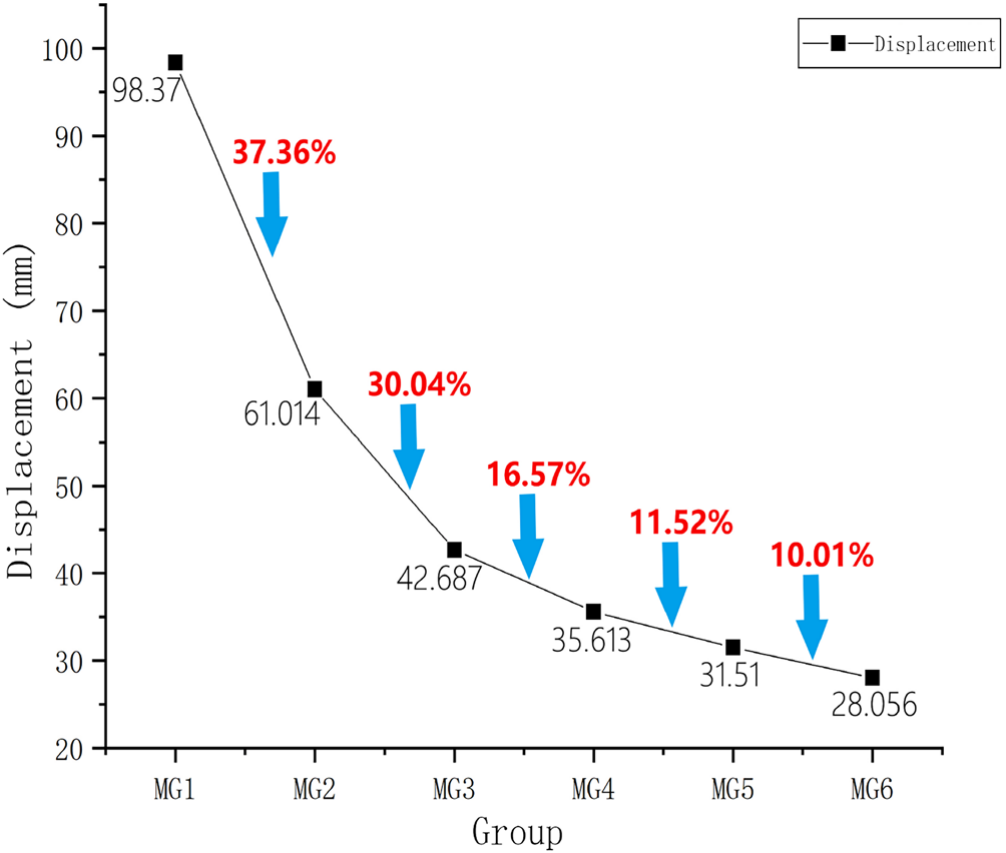
Displacement-change curves for the nine-pile models with different disc overhang diameters under the same load.

The load–displacement data were extracted for the six nine-pile models with different disc overhang diameters from ANSYS finite element simulation software, and the corresponding load–displacement curves were constructed, as shown in Fig. 19. The pile displacements were 8,550 kN when MG1 reached the ultimate load state. The displacement-change curves for the models with different disc overhang diameters under the same load are presented in Fig. 20.

Figures 19 and 20 show that the trends for the nine-pile models were approximately the same as those for the six-pile models. As the disc overhang diameter increased, the vertical displacement of the pile body decreased and the uplift bearing capacity increased. Initially, the load–displacement curve decreased slowly; however, the rate of decrease became steeper during the middle stages, and there was a rapid decrease in the later stages. This indicates significant soil interactions in the nine-pile model.

The pile displacements were extracted at 8550 kN when group MG1 reached the ultimate load state, as shown in Fig. 20. The displacements of the pile tops in group MG1 were 98.37 mm. In comparison, the displacement of the pile tops in group MG2 was 37.36% lower than that in group MG1. Furthermore, the displacement of the pile tops in group MG5 was 11.52% lower than that in group MG4, and the displacement in group MG6 was 10.01% lower than that in group MG5. As a result, the displacement-change curve shown in Fig. 19 was produced. As the disk overhang diameter increased, the displacement at the top of the pile body gradually decreased, and the uplift bearing capacity gradually increased. However, the increase in uplift bearing capacity was not linear. When the disk overhang diameter was greater than 1.75*d* (i.e., groups MG5 and MG6), there was little change in the soil displacement near the piles, and the uplift bearing capacity of the pile groups did not increase significantly. Therefore, the disc overhang diameter should be 1.5–1.75*d*.

Analysis of the load–displacement curves for the four-, six-, and nine-pile models showed that the uplift bearing capacity increased as the disc overhang diameter increased. However, this change is not linear, and the uplift bearing capacity increases most rapidly when the disc overhang diameter is 1.5–1.75*d*. Considering factors such as bearing capacity, settlement control, cost, and material savings, the optimum disc overhang diameter is 1.5–1.75*d*.

The load–displacement curves for the double-, four-, six-, and nine-pile models were further analyzed to investigate the effects of the pile group on the uplift bearing capacity.

### Load–displacement curves for the double, four-, six-, and nine-pile models

**Figure 21:**
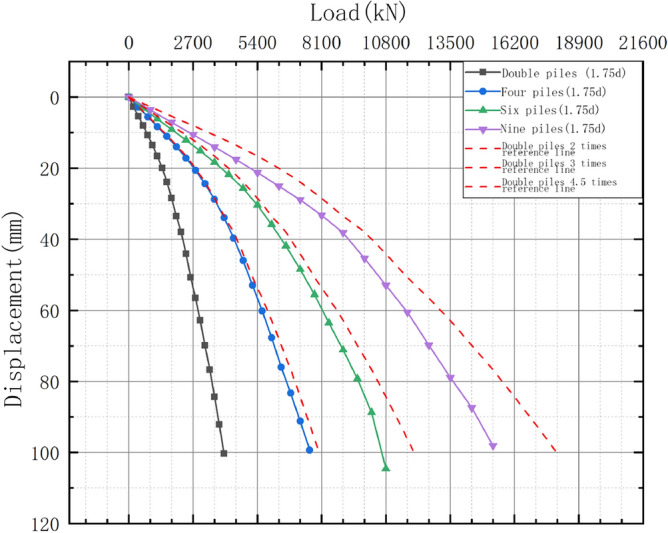
Load–displacement curves for the double-, four-, six-, and nine-pile models.

Using ANSYS finite element simulation software, extract the top load displacement curve data of double-, four-, six-, and nine-pile models with a disk overhang diameter of 1.75d, and draw the load displacement curve as shown in Fig. 21.

As can be seen, the load–displacement curves for the six- and nine-pile models differ from those of the four-pile model. During the loading process, the pile body curves and reference lines did not coincide, indicating that soil interactions occurred during the initial loading stage; therefore, they could not be considered as single piles.

Meanwhile, further analysis of Fig. 21 shows that the distance between the six-pile load–displacement curve and the reference line (three times the double-pile line) increased as the vertical tension increased, indicating that the pulling capacity of the six-pile model was less than three times that of the double-pile model. Therefore, the pulling capacity of each pile in the six-pile model is lower than that in the double-pile model. Furthermore, the difference between the pulling capacities of the six-and three times the double-pile models gradually increased as the load increased.

Extract the ultimate load values of double piles, six piles, and nine piles using ANSYS finite element simulation software.The failure loads of the six- and nine-pile models were approximately 2.741 and 3.894 times those of the double-pile model, respectively. Therefore, the increases in the pile number and uplift capacity were not linear, and the uplift capacity was reduced owing to the pile group effect.

### Summary of vertical bearing behavior of four, six and nine piles

Using ANSYS finite element simulation software, extract the ultimate load values of single pile, double pile, four pile, six pile, and nine pile at this time, as shown in Table 2:

**Table 2:** Comparative data of bearing capacity of four-pile, six-pile and nine-pile.

Number of piles	1	2	4	6	9
Ultimate load (kN)	1997.6	3855.38	7386.9	10567	15010.47
Average load of single pile (kN)	1997.6	1927.69	1846.73	1761.17	1667.83

From the data in Table 2, it can be seen that as the number of piles increases, the ultimate load value of the pile body gradually increases, and the overall bearing capacity of the pile body increases. However, the average load value of a single pile gradually decreases, indicating that increasing the number of piles does not increase the bearing capacity exponentially. The average bearing capacity of a single pile will decrease with the increase of the number of piles, and the more the number of piles increases, the greater the reduction in bearing capacity. In practical engineering, increasing the number of piles will also increase construction costs. Therefore, increasing the quantity alone cannot be the main way to improve the bearing capacity. It is necessary to comprehensively consider factors such as the overhanging diameter of the disc, the spacing between piles, the number of discs, and the number of piles, in order to achieve the goals of bearing capacity, settlement control, lowest cost, and material conservation.

## Conclusion

Through studying the uplift failure mechanisms and bearing performances of the NT-CEP pile groups with different disc overhang diameters, the following conclusions were obtained.

1. As the disk overhang diameter increased, the uplift-bearing capacities of the pile groups increased; however, this relationship was not linear. Considering factors such as the bearing capacity, settlement control, cost, and material savings, the maximum disc overhang diameter should be 1.5–1.75 times the pile diameter.
2. The pile group effect was investigated considering a constant disc overhang diameter. In the six-pile model, the tensile strengths of the corner piles were higher than those of the side piles. For the nine-pile model, the tensile strength of the corner piles was greater than that of the side and center piles. This indicates that the pile group effect depends on the positions of the piles.
3. Owing to the pile group effect, the pulling capacity of the pile groups did not increase linearly as the number of piles increased. The average pulling capacity of a single pile in the pile group was lower than that of a single pile, which reduced the pulling capacity of the pile group. As the number of piles increased, the pile group effect became more pronounced, and the reduction in carrying capacity increased.

This investigation of the effects of the disc overhang diameter on the load performance of NT-CEP pile groups provided an optimal range for the disc overhang diameter. Moreover, this study can be used as a reference when determining the reduction coefficient and as a calculation model for the uplift bearing capacity of NT-CEP pile groups with different disc overhang diameters. Hence, this study provides a reliable theoretical basis for practical engineering applications using NT-CEP pile groups.

## Data Availability

Data are contained within the article.

## References

[1] Ma H, Wu Y, Tong Y, Jiang X (2020). Research on bearing theory of squeezed branch pile. Adv. Civ. Eng..

[2] Zhang M, Xu P, Cui W, Gao Y (2018). Bearing behavior and failure mechanism of squeezed branch piles. J. Rock Mech. Geotech. Eng..

[3] Gao X, Wang J, Zhu X (2007). Static load test and load transfer mechanism study of squeezed branch and plate pile in collapsible loess foundation. J. Zhejiang Univ. Sci. A.

[4] Ju Y, Chen YF (2018). Experimental study for the bearing capacity calculation of concrete expanded plates in squeezed branch piles. Mater. Test..

[5] Tang ST, Chen LH (2011). Field test of dx pile group. Adv. Mater. Res..

[6] Do DH, Pham TA (2018). Investigation of performance of soil-cement pile in support of foundation systems for high-rise buildings. Civ. Eng. J..

[7] Wang H, Ran Z, Yan J (2022). Design and application of expanded pile in bridge super-large diameter pile foundation. Jiangxi Build. Mater..

[8] Wang H, Jin X (2022). Application of construction technology of super-large variable diameter consolidated disc expanding pile in confined space under silty sand geology. Eng. Const. Des..

[9] Qian YM, Chen Y, Xv LN (2022). Influence of the α Angle of Flexible Concrete Expanded-Plate Piles on the Bearing Capacity under Horizontal Load. Adv. Mater. Sci. Eng..

[10] Qian YM, Zhai RZ (2015). Analysis of the effect of the space of the bearing plate on the uplift bearing capacity of the concrete plates-expanded pile. Open Civ. Eng. J..

[11] Qian YM, Wang X, Wang RZ (2015). Research on feasibility of controlling crack resistance of the concrete expanded-plates pile under vertical tension. Open Const. Build. Technol. J..

[12] Fattah MY, Al-Obaydi MA, Al-Jalabi FA (2018). Effect of number of piles on load sharing in piled raft foundation system in saturated gypseous soil. Int. J. Civ. Eng. Technol. (IJCIET).

[13] Hadi DH, Waheed MQ, Fattah MY (2021). Effect of pile's number on the behavior of piled raft foundation. Eng. Technol. J..

[14] Qian YM, Dong Y, Tian W, Jin YJ (2019). Theoretical analysis on the influence of the slope angle of plate for the failure mechanism of the concrete expanded-plates pile applied for oceanographic engineering under the horizontal force. J. Coast. Res..

[15] Yu H (2023). Study on the effect of disc cantilever diameter on the uplift bearing performance of double piles of concrete expanded pile. Build. Struct..

[16] Zhu HT (2008). Application of reinforced concrete element SOLID65 in ANSYS software. Heilongjiang Sci. Technol. Inf..

[17] Lv T, Zhao J, Xue L (2010). The influence of modeling methods on the meshing when solid element was used in ANSYS - Taking the design by analysis of dry welding experiment module as an example. Appl. Mech. Mater..

[18] Xing J, Li J (2014). The modeling method and the mesh division of the ANSYS. China Water Transport (Academic).

[19] Song YJ, Hu W, Wang DS (2011). Analysis of compacted pile squeezing effect based on modified Cambridge model. Rock Soil Mechan..

[20] Li, Q. L. Journal of Shanghai Dianji University, 05, 28–30. (2006)

